# Living with cystic fibrosis during the COVID-19 pandemic: a social connectedness perspective

**DOI:** 10.1080/17482631.2022.2062820

**Published:** 2022-04-19

**Authors:** Maggie Harrigan, Kellie Bennett, Siobhain Mulrennan, Melanie Jessup

**Affiliations:** a UWA Medical School, The University of Western Australia, Perth, Western Australia, Australia; b Cystic Fibrosis Research Unit, Institute for Respiratory Health (IRH), Nedlands, Western Australia, Australia; c Cystic Fibrosis Clinic, Department of Respiratory Health, Nedlands, Western Australia, Australia; d School of Nursing, Midwifery and Social Work, The University of Queensland, Brisbane, Queensland, Australia

**Keywords:** Cystic Fibrosis (CF), qualitative, social connectedness, COVID-19, belonging, resilience, social support, mental health, chronic illness, Western Australia

## Abstract

**Purpose:**

This study explores the concept of social connectedness for adults with Cystic Fibrosis (CF), generally and during the onset of the COVID-19 pandemic, to help inform contemporary CF healthcare. Social connectedness is an essential component of belonging and refers to an individual’s sense of closeness with the social world. Unique disease factors make exploration of social connectedness pertinent, added to by COVID-19, with the CF population potentially facing increased risk for severe illness.

**Methods:**

Seventeen adults with CF in Western Australia undertook interviews, with findings categorized as overarching themes.

**Results:**

In a general sense, participants described social connectedness challenges caused by CF, despite which they reported meaningful connections that benefits their mental and physical health. Within a COVID-19 specific context, participants demonstrated resilience in the face of adversity, highlighted the importance of empathy in relation to the pandemic, and described how social support is both an outcome and enhancer of social connectedness.

**Conclusions:**

This study contributes to limited social connectedness literature within CF and chronic illness in general, highlighting the importance of social connectedness awareness raising, assessments and interventions in CF healthcare inside and outside the COVID-19 pandemic.

## Introduction

Kohut’s (1984) belonginess theory states that humans have a fundamental need for quality and meaningful social relationships in order to optimize functioning in life (Kohut, 1984). Lee and Robbins (1995) later expanded upon this theory by conceptualizing social connectedness, defined as one’s subjective awareness of close social relationships (Lee & Robbins, 1995). This study will focus on key dimensions of social connectedness in line with belongingness theory; namely emotional closeness, feeling bonded, being understood, and a sense of belonging (Kohut, 1984; Lee & Robbins, 1995). Underpinned by Kohut’s (1984) belongingness theory, strong feelings of social connectedness enhance an individual’s mental and physical health (e.g., Begun et al., 2018; Deindl et al., 2016; Lee et al., 2001). Within healthcare, empirical evidence indicates that weak social connectedness represents a serious risk factor for several chronic illnesses (Larrabee Sonderlund et al., 2019). The limited research that exists in a physical chronic illness context indicates that low levels of social connectedness are a strong predictor of depression, anxiety, and poorer general health (Hatchcock, 2012).

CF is the most common life-threatening genetic condition in Australia (Cystic Fibrosis Australia (CFA), 2019). CF is a multisystem condition that results in progressive chronic lung disease (Cosgriff et al., 2020) and can cause other comorbidities, such as diabetes, liver disease and malnutrition (Cosgriff et al., 2020). Knowledge and treatment of CF has had an astounding evolution, from disease recognition in 1938, when most babies born with CF died before their first birthday, to present day, where Australians with CF can expect to live well into adulthood (Cystic Fibrosis Australia (CFA), 2019).

There are only two CF specific social connectedness studies, both of which have identified the importance of social connectedness for people with CF and recognize the need for further research (Francis et al., 2020; Toth, 2016). Social connectedness within CF is particularly relevant given various pertinent disease factors. For example, infection control guidelines preclude people with CF from meeting each other in-person and recommend avoidance of every day respiratory infections (Cystic Fibrosis Foundation (CFF), 2020; Saiman et al., 2014), there is a demanding daily CF treatment regime (Sawicki et al., 2009), and there is a relatively high prevalence of anxiety and depression symptoms within the CF population (Quittner et al., 2014a). Social connectedness is also a particularly relevant concept to explore within a CF context during a health pandemic. People with CF might be at an increased risk for severe illness from this virus compared to healthy age matched individuals (Centers for Disease Control and Prevention (CDC), 2021). It follows that people with CF might be at greater health risk from in-person contact, and thus significantly affected by the social distancing restrictions implemented across Australia during early 2020.

The aim of this study is to explore the concept of social connectedness within the unique context of adults with CF and during the early onset of the COVID-19 pandemic, providing a window into lived experiences to help inform contemporary CF healthcare.

## Background

### Belongingness theory

Kohut’s (1971, 1977) psychoanalytic self-psychology theory views the self as the centre of a person’s psychological world, and the relationship between the self and other people as vital for self-expression. Specifically, the self is the centre of experience and has a fundamental need for belongingness (Kohut’s, 1971; Kohut, 1977). Belonginess theory expands upon psychoanalytic self-psychology theory, holding that humans have a fundamental and unescapable need to make and continue strong interpersonal bonds to optimize functioning in life (Kohut, 1984). Belongingness theory states that it is not just contact with others that satisfies our innate need for belonging, but the sense of quality and meaning we derive from this contact, a strong sense of which will enhance mental and physical health (Kohut, 1984). Lee and Robbins (1995) later conceptualized this sense, coining the term social connectedness.

### Social connectedness

The concept of social connectedness is an important facet of belonging (Lee & Robbins, 1995). Social connectedness refers to an individual’s sense of closeness with the social world (Ang et al., 2015; Lee & Robbins, 2000; Satici et al., 2016), tapping into aspects of belongingness that Kohut (1984) described as feeling *“human among humans”* (p. 200). At the heart of social connectedness is an individual’s perceived feelings of social bonding, meaningful connection, and interpersonal relatedness (Grieve & Kemp, 2015; Lee et al., 2001). Social connectedness is how we view and feel about ourselves in relation to other people (Lee & Robbins, 2000; Sinclair & Grieve, 2017). The sense of interpersonal closeness derived from social connectedness includes both proximal relationships such as family, and distal relationships such as general society (Lee & Robbins, 1998). Individuals who have a strong sense of social connectedness tend to feel a sense of closeness to, and understanding by, other people (Lee et al., 2001).

Social connectedness has been extensively researched within a wide range of non-healthcare settings, such as older adults, homeless youth, college students and rural communities (e.g., Begun et al., 2018; Caxaj & Gill, 2017; Collins et al., 2017; Deindl et al., 2016; Lee et al., 2001). This research has found that social connectedness is positively related to mental and physical health (e.g., Begun et al., 2018; Caxaj & Gill, 2017; Collins et al., 2017). In healthcare, research indicates that weak social connectedness represents a serious risk factor for several chronic illnesses, such as cardiovascular disease, diabetes, various cancers, and mental health (Holt-Lunstad et al., 2010; Larrabee Sonderlund et al., 2019). The quality and quantity of an individual’s social relationships is viewed a predicter of morbidity and mortality (Holt-Lunstad et al., 2010). Subsequently, healthcare researchers have called for the inclusion of social connectedness assessments in routine medical settings (Holt-Lunstad et al., 2010; Larrabee Sonderlund et al., 2019). Within a mental illness setting, social connectedness is deemed an essential dimension of personal recovery (e.g., Leamy et al., 2001; Pahwa et al., 2019; Sweet et al., 2018). Social connectedness research within physical chronic illness is limited, but considered essential to combatting the social isolation that can often accompany chronic illness (Stenberg & Furness, 2017). Low levels of social connectedness are also considered a strong predictor of depression, anxiety and general health within this population (Hatchcock, 2012). In an unfortunate and complex interplay, physical chronic illness has been found to be a disrupter of social connectedness (Person et al., 2007; Soleimani et al., 2014) and low levels of social connectedness an exacerbator of the negative consequences of living with a physical chronic illness (Person et al., 2007).

### CF and social connectedness

There are currently only two studies of social connectedness within a CF setting. First, Toth (2016) identifies social connectedness as something that young people with CF value, desire, and develop despite disease and treatment burden (Toth, 2016). Second, Francis et al. (2020) evaluates the use of a smartphone application to improve social connectedness and well-being. This study focuses on adolescents and the useability of the application, calling for future research that tests the effectiveness of such technologies on social connectedness and well-being (Francis et al., 2020).

Pertinent disease factors make social connectedness within CF an important research area. First, infection control guidelines precluding people with CF meeting each other in-person emerged during the nineties due to increasing concern regarding transmission of pathogens between people with CF (Saiman et al., 2014). Prior to this time, many people with CF were readily socializing in-person, using a range of group support settings to facilitate relationship development and sharing of experiences with others with CF (Muther et al., 2018). This type of in-person contact is known to be beneficial to people in many disease groups, by enhancing social connectedness and validation of experience (Kirk & Milnes, 2015). Infection control guidelines introduced in the nineties therefore added a unique social connectedness barrier for those with CF. Additionally, guidelines also recommend that people with CF avoid every day respiratory infections, such as the common cold, because contamination could have more serious consequences compared to someone without CF (Cystic Fibrosis Foundation (CFF), 2020). Infection control, in-person contact restrictions and social isolation are therefore issues the CF community have been managing long before the arrival of COVID-19, and part of this management has involved using online communication tools (e.g., Grossoehme et al., 2012; Moola, 2018; Quittner et al., 2016). The demographics of the adult CF community include a significant number of people who have grown up with or are competent with online communication (Dale et al., 2016). Consequently, the online CF community is strong, with many CF health professionals, people with CF and the family members of those with CF embracing a vast array of online communication tools, such as social media, to promote communication, information sharing, connection and support (Strekalova, 2016; Dale et al., 2016; Jeffrey et al., 2020).

Second, CF has a demanding treatment regime, which extends life (O’Toole et al., 2019; Sawicki et al., 2009). Treatments involve physiotherapy, enzyme replacement capsules, antibiotic therapy, aerosol mist inhalations, specific dietary requirements and exercise (Cystic Fibrosis Australia (CFA), 2019). Many of these treatments are daily and preventative, but there are also further treatments and hospitalizations required during disease exacerbations (O’Sullivan & Freedman, 2009). Rigorous CF treatment requirements have the potential to impact lifestyle, relationships, and the ability to socialize, and thus are likely to impact social connectedness (Quittner et al., 2016; Sawicki et al., 2009).

Lastly, symptoms of anxiety and depression are between 200 to 300% higher in individuals with CF than those without the disease (Quittner et al., 2014a). Outside of a CF context, social connectedness has been negatively associated with these symptoms (Begun et al., 2018; Lee & Robbins, 1998: Malaquias et al., 2015). Understanding factors that impact mental health is particularly important within a CF setting, where suboptimal mental health is associated with poorer physical health outcomes (Ploessl et al., 2014; Snell et al., 2014), poorer adherence to medical treatment (Hilliard et al., 2015; Smith et al., 2010), and detrimental economic consequences as a result of frequent hospitalizations and higher healthcare costs (Snell et al., 2014). Alarmingly, new research also associates depression with increased mortality for adults with CF (Schechter et al., 2021).

### COVID-19

COVID-19 is part of a family of viruses that cause respiratory infections (Australian Government, Department of Health, 2020). In the initial stages of the COVID-19 pandemic, the health risks to people with CF were completely unknown and many countries considered this population to be highly vulnerable to the virus (Cosgriff et al., 2020). Since this time, initial research into the effects of COVID-19 in the CF population suggest outcomes similar to the general population (Cosgriff et al., 2020; McClenaghan et al., 2020). A more severe clinical course however may be associated with older age, CF-related diabetes, lower lung function in the year prior to infection, and having received an organ transplant (McClenaghan et al., 2020). Whilst outcomes are better than initially feared overall, the Centres for Disease Control and Prevention (CDC) state that having CF might increase the risk of severe illness from COVID-19 compared to healthy age matched individuals (Centers for Disease Control and Prevention (CDC), 2021), and that those in this cohort who have had an organ transplant are at increased risk (Centers for Disease Control and Prevention (CDC), 2021). Given that COVID-19 is a contagious disease that can be transmitted person-to-person, it follows that people with CF might be at greater risk from in-person contact (Centers for Disease Control and Prevention (CDC), 2021).

COVID-19 presents significant social and psychological challenges for the general population (Brooks et al., 2020), but particularly for those living with a chronic illness who face an additional layer of complexity to life (Amja et al., 2021; Druss, 2020). People in general with underlaying mental and physical illness are at highest risk of social isolation and consequent adverse psychological effects due to the COVID-19 pandemic (Druss, 2020; Razai et al., 2020). CF specific research highlights the importance of trusting human relationships between health professionals and those living with CF, and encourages the use of online communication amidst COVID-19 to share information and promote psychological health (Nobili et al., 2021). Interestingly, preliminary studies demonstrate that adults with CF have similar levels of psychological distress during COVID-19 compared to the general population (Ciprandi et al., 2021; Haversman et al. 2020). One may speculate that this could be owing to resilience resulting from life-long experience coping with infection control (Ciprandi et al., 2021). Nonetheless, COVID-19 has undoubtedly created psychological distress for those with CF and exploring social connectedness within this setting is opportune.

## Materials and methods

This study took place in Western Australia, where the Government had declared both a State of Emergency and a Public Health Emergency on 15 March 2020 in response to COVID-19. This study commenced on 6 May 2020, when there were 551 confirmed cases of COVID-19 and nine COVID-19 deaths in Western Australia (Government of Western Australia, Department of Health, 2020). The study commencement date marked one week of no new COVID-19 cases reported in Western Australia. Due to the contagious nature of COVID-19 and lack of vaccination at that time, the Western Australian government implemented a range of stringent control measures early into the pandemic to limit the risk of COVID-19 spreading throughout the community (Western Australia Government, 2020a). These “*go hard, go early”* (ABC News, 2021) measures included all West Australians staying at home with rare exemptions, the closure of recreation facilities and non-essential businesses, gatherings excluding household members limited to two people, home-schooling, home-based childcare, and restricted intrastate and interstate travel (Western Australia Government, 2020a). The first phase of easing contact restrictions began on 27 April 2020, just prior to study commencement (Western Australia Government, 2020b). The second phase of easing contact restrictions commenced on 18 May 2020, just prior to the study ending on 23 May 2020 (Western Australia Government, 2020b).

### Participants

Following approval from The University of Western Australia Human Research Ethics Office (RA/4/20/6105) in April 2020, participants were recruited by the Institute for Respiratory Health (IRH). IRH is a research institute based in Western Australia dedicated to investigating respiratory disease. IRH hold a database of contact details for people with CF who have been involved in previous studies. An IRH recruitment officer selected people at random by telephoning every fifth person on the database. To participate in the study, people had to be at least 18 years old, with a CF diagnosis, English speaking (this did not exclude anyone approached to take part in the study), and medically stable (not a hospital in-patient) at time of interview. The study had strict time limitations on the recruitment and interview phase, to ensure a consistent COVID-19 context during data collection. All participants had to be recruited and interviewed within a four-week timeframe, when they were all dealing with similar COVID-19 rates, information and restrictions. This timeframe allowed for thirty-two people to be contacted by the IRH recruitment officer, with 21 (66%) people voicing interest in the study and openness to further information. Further contact was attempted with 20 of these people within time constraints, 17 of which were recruited as they scheduled an interview and provided informed written consent within the study timeframe. Participants consented to the publication of information provided. All participants were provided with information on where to access mental health support post interview if required, however no known adverse effects were caused by participation.

### Interview

The aim of this study was to explore the concept of social connectedness within the unique context of adults with CF, during the early onset of the COVID-19 pandemic. This was achieved through a constructionist perspective to research, where the lived experience of social connectedness was explored, with the view that all knowledge is contingent upon human practices (Crotty, 1998).

Semi-structured interviews were carried out with 17 adults with CF. A deductive approach was adopted, using the constructs of belongingness theory and social connectedness to inform the development of interview questions. An inductive approach was then used, gathering lived experiences, examples and observations to guide findings (Crotty, 1998). Semi-structured interviewing enabled openness and flexibility for participants to talk about what they deemed important, while an interview schedule supported focus and analysis. Prior to interview, participants were provided with an explanation of social connectedness that focused on key dimensions of the concept in line with belongingness theory; specifically that it is a feeling of emotional closeness, feeling bonded, being understood, and a sense of belonging to people in your life that you know, and the world in general (Kohut, 1984; Lee & Robbins, 1995). The Social Connectedness Scale—Revised is a reliable and validated quantitative tool to measure levels of social connectedness (Lee et al., 2001), and was initially drawn upon in question development. The focus of this study, though, was to gather qualitative data surrounding experiences of social connectedness.

Initial interview questions were broad (e.g., How are you feeling about the COVID-19 situation?), followed by more specific questions around contact with people and the meaning of these contacts (e.g., Has COVID-19 changed the amount and means of contact you are having with people? How do you feel family and friends have understood what COVID-19 means for you?). Questions addressed proximal and distal relationships e.g., family and friends and the general public. Final questions explored value placed upon social connectedness (e.g., Do you think social connectedness is important, and if so, why?). Due to COVID-19 transmission concerns online encrypted videoconferencing was used, allowing audio-visual queues and real-time conversation, and offering time and travel cost advantages to participants (Irani, 2019; Salmons, 2012). Participants were also given the choice of being interviewed via telephone to promote socioeconomic inclusivity. Interviews lasted approximately 40 minutes, were audio recorded, transcribed and anonymized.

### Data analysis

Data analysis was in-depth and not subject to the same time constraints placed on data collection. Data was analysed by hand through microscopic examination, analysing interview transcripts line-by-line in order to identify themes (Strauss & Corbin, 1998). The Miles et al. (2013) transcendental realism framework for thematic analysis was employed, consisting of three main and concurrent components. First, data was reduced through editing and segmenting, to identify themes, clusters and patterns and conceptualize and explain the data. Second, repeated and iterative data displays were used to organize, compress and assemble voluminous and dispersed information, showing what stage analysis had reached, and guiding further analysis. Last, conclusions were drawn and verified by the researcher team through a concurrent process, which was sharpened as analysis progressed. Despite in-depth analysis, it is assumed that theoretical saturation was not met by this study, given time constraints placed on data collection.

### Findings

All 17 participants opted for online videoconferencing, with no practical challenges when scheduling or conducting interviews. All participants voiced familiarity with online communication. Participant demographics are detailed in Table I.

**Table I.** Participant demographics

Demographic variable	Response	Number	Percentage (%)
**Sex**	Female	7	41
	Male	10	59
**Age**	19–29 years old	3	18
	20–39 years old	6	35
	40–49 years old	5	29
	50–59 years old	3	18
**Residence**	Metro	12	71
	Regional	5	29
**Lives with**	Someone	15	88
	Alone	2	12
**Relationship Status**	Married	11	65
	Long-term relationship	4	24
	Single	2	12
**Lung Function**	30–50%	4	24
	51–70%	5	29
	71–90%	4	24
	91% +	4	24
**Employment/Education Status**	Employed	10	59
	Unemployed	3	18
	Employed and Studying	4	24

**Figure 1.**
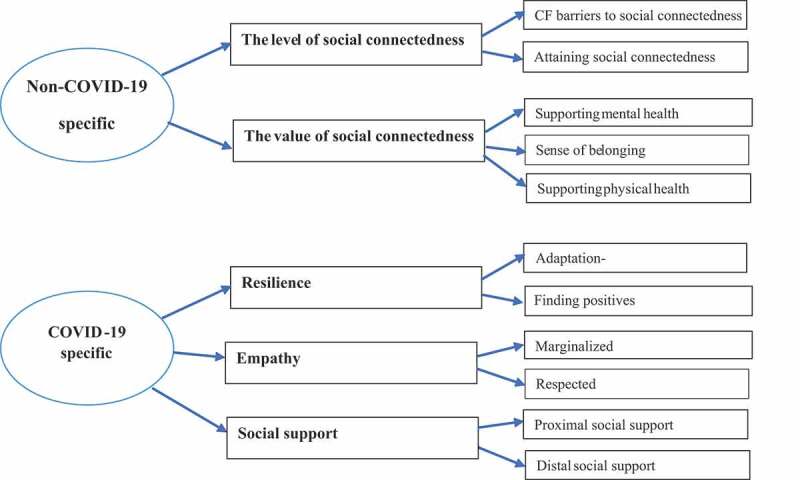
Overview of prominent themes.

Findings are categorized by overarching and prominent themes, the first two of which relate to a general, non-COVID 19 specific context: (1) the level of social connectedness and (2) the value of social connectedness. The last three themes relate specifically to a COVID-19 context: (1) resilience, (2) empathy and (3) social support (see, Figure 1).

### Non-COVID-19 specific: Theme 1—The level of social connectedness

The level of social connectedness refers to how adults with CF connect to family and friends without CF in a general sense not specific to COVID-19. This theme describes the social connectedness challenges created by CF and how participants have overcome these challenges. Interestingly, the small number of participants who did not experience these difficulties described themselves as relatively healthy and did not identify strongly as a person with CF. Two sub-themes are derived.

### Sub-theme—CF barriers to social connectedness

The majority of participants reported experiencing a lack of understanding regarding their CF or CF creating barriers for social connectedness within proximal relationships. This sub-theme centred around the complex and chronic nature of the disease. Several participants spoke about people struggling to understand CF, given the lack of outward symptoms. One participant articulated this by saying:

When I’m meeting people I know, and they constantly say, “Oh, you look well,” because one thing with CF is you don’t look necessarily how you feel.

Some participants described the long-term nature of CF, involving cyclical periods of being well and unwell, and subsequent implications on social participation and connectedness. One participant highlighted the long-term nature of CF, saying:

CF is a layered complex thing. Some people would like to know just the fluffy bits of it, and they don’t want to know all the hard side and it overwhelms them … some people just don’t understand that it is a long-term thing.

Several participants talked about CF impacting peer relatability in physical and emotional senses. One participant commented on emotional relatability, saying:

Meeting the transplant team or being quite sick at one point, even stuff we went through trying to have a family … In some ways we’ve had to grow up a lot … when we meet new people and they might be moaning … I find maybe I don’t have as much patience … so I would be in their company, but perhaps not find the need to develop a really deep friendship.

### Sub-theme—Attaining social connectedness

Despite CF creating barriers to social connectedness, all participants described relationships with family or friends without CF providing them with a sense of social connectedness. Within these relationships, participants often experienced understanding regarding their CF and identified feelings of emotional closeness, bonding and belonging. One participant expressed this by stating:

My friends know that my lungs aren’t good. They know what my limitations are … they ride the good times and the bad times with me.

Almost all participants identified an inner friendship group; a key friend or group of friends with whom they felt the deepest sense of social connectedness. One participant articulated this by remarking:

I think because my closest friends are my oldest friends, there’s never been a problem with connectedness, because they’ve known from the beginning about CF, and they’ve lived life with me as I go along. They ride with me.

Interestingly, some participants identified CF as enhancing their sense of social connectedness, particularly with parents who supported their health from childhood. One participant stated:

I do appreciate my parents for everything they did for me when I was first born. There was a lot of medical care, a lot of trips to the hospital … As I got older … I appreciated that, and that made me close to my parents.

### Non-COVID-19 specific: Theme 2—The value of social connectedness

All participants reported social connectedness as a fundamentally important part of life in a general context, including descriptions of social connectedness as an innate human need. One participant stated:

I think social connectedness is absolutely massive … . We’re social beings. We’re humans … . We all have that social need.

Three sub-themes detail reasons participants deemed social connectedness important.

### Sub-theme—Supporting mental health

The majority of participants felt that social connectedness supports their mental health. Participants described how social connectedness results in an ability to communicate with people, particularly in times of emotional difficulty, and that this supports their mental health. One participant demonstrated this by saying:

Social connectedness is important for mental health, no one wants to feel alone, and like they can’t go to anyone. Having someone you can talk to about something that’s troubling you, offer you advice … I think it’s so important … . When I’ve had my worst moments of mental health is when I’m not feeling that kind of connection to friends.

Furthermore, participants highlighted how social connectedness diminishes feelings of loneliness, which supports their mental health. Participants described how social connectedness provides them with a level of mental comfort by preventing them having to manage emotional difficulties alone. This was highlighted by one participant, who stated:

I think social connectedness is definitely important. Even in a general sense, just to tell someone about your day and struggles you might be having, and then you find out they’re having the same struggles. There’s certain things that you can just talk through and even just talking it through with someone, you then have a light bulb moment and go, “Actually, you haven’t told me anything, but just by talking to you, I’ve just realized another two three things about it.” So, I think it’s very important.

### Sub-theme—Sense of belonging

A number of participants described social connectedness providing them with a sense of belonging. Feelings of closeness and connection to a group of people were associated with this sense of belonging. One participant demonstrated this by stating:

I had a particular group of cousins when I was younger, I felt more like they were like my siblings. We do a lot of things together, and we still keep in touch and do that sort of stuff. And when they’re doing things, they invite me as if I’m one of them. So I feel connected to them, close to them.

Participants detailed feelings of comfort and acceptance derived from a sense of belonging. Feelings of fitting-in, inclusivity and being part of something were also particularly important to participants. One participant commented:

You hear people say that humans are pack animals and really feel like you need somewhere to belong. And if you don’t have a place to belong, it can be depressing, not having a sense of belonging to groups. I think that part of social connectedness is the key thing for me. It’s having a group that I belong to and where I feel comfortable that I belong.

### Sub-theme—Supporting physical health

Several participants stated that social connectedness supports their physical health, by providing them with motivation to undertake CF treatments, such as exercise and medications. One participant expressed this by saying:

If you don’t have social connectedness, it’s like you’re by yourself. Sometimes, external motivation to exercise is a great support.

A sense of togetherness underpinned the motivation to undertake treatment, where participants did not feel alone in managing treatment. This is highlighted by one participant who commented:

Mum will stay up with me while I do my treatment, even though I’m in my 30s. Mum will just stay up because that’s what we did when we were little … So I never miss my treatment.

### COVID-19 specific: Theme 1—Resilience

The overarching theme of resilience is derived from the majority of participants demonstrating features of resilience during COVID-19. Understandably, participants described fear and stress resulting from COVID-19. Most participants described a high level of in-person contact prior to COVID-19, which reduced during the pandemic. This reduction involved not seeing family and friends in-person, not partaking in social activities and adapted work practices. Despite these challenges however, participants impressively described an ability to adapt and identify “silver linings” amidst such adversity. Two sub-themes add further detail.

### Sub-theme—Adaptation

A large majority of participants detailed access and skills in using a wide variety of online communication platforms prior to the pandemic. Participants described honing and increasing this online communication as a means of adapting to COVID-19 contact restrictions. This adaptation enabled them to maintain social connectedness, allowing contact and emotional closeness with people despite not seeing them in-person. This is highlighted by one participant who commented:

I grew up with computers all my life, so I have had the internet from quite an early age. I’ve always had connectedness on that level as well … Over the last five years, a lot of the chatter is at either a text level or messenger level, so there’s already been that transition, but certainly it’s migrated more to online than it did before, even to the point where I’m catching up with my friends online probably more so than I did in real life at the moment.

### Sub-theme—Finding positives

Almost all participants expressed heightened fear in the very early days of the pandemic when little to nothing was known about the impact and timeframes associated with COVID-19. Despite some remaining uncertainty and fear, a large number of participants identified things which they feel grateful for, articulating positives amidst COVID-19. One participant commented:

It definitely has its good points, because it lets you reflect on what you’ve got … .now I do a workout, spend more time playing with my daughter, the house improvements.

Another said:

Actually coronavirus worked in my favour, really. It was probably the push I needed to take time off work.

### COVID-19 specific: Theme 2—Empathy

This theme refers to perceptions of empathy specific to COVID-19, particularly surrounding contact restrictions. Many participants shared mixed experiences, providing examples of when they felt misunderstood and marginalized, and conversely when they felt understood and respected. Reassuringly, the overall picture was a positive one, with participants predominantly experiencing empathy.

### Sub-theme—Marginalized

Many participants perceived a lack of understanding from family, friends, or the general community regarding what the pandemic meant for them, resulting in feeling different or unimportant. A number of participants described experiences where they felt family and friends did not understand the seriousness of the pandemic, particularly the need for certain contact restrictions. This is demonstrated by one participant who remarked:

I told a family member off a few times, because he went out on the weekend … . and I kept saying, “You’re not supposed to be doing this.” But I suppose for them it was like, well if I get it it’s no big deal … . They really continued on as per normal really. I wasn’t happy about that.

Interestingly, a small number of participants felt over-protected in relation to contact restrictions, with one participant describing discomfort at being made to feel different, stating:

I guess my friend is trying to show that he cares by saying that he’d still like me to be isolated and look after myself, but then he thinks that everyone else should be going back to work and not being sensitive to the excluding feeling that gives … that makes me feel different which I don’t get very often … .Stay home with all the old people.

Another participant expressed frustration that friends did not appreciate that infection control was not a new concept for them, stating:

Friends are a bit overprotective and annoying … I’ve pretty much read them the riot act because it’s my life. I deal with this every season that flu comes along, so calm down.

Some participants described a lack of community understanding. They felt that the community did not want interruption to their usual lifestyles and were not supportive of contact restrictions. This eroded a sense of community and togetherness. One participant highlighted this by stating:

I’d say COVID-19 has maybe brought out some things where I’m struggling with people … just going, “This is just a flu. Let’s all get back to normal, and if a few people die, they’ve probably got other issues.” … I feel like saying, “Well, who you’re talking about is me, because I’m one of those people.” … All they worry about is the interruption to their life … not looking at it as a community thing.

Interestingly, a few participants expressed concerns regarding how their CF-related coughing will be perceived by the general public during or after the pandemic, with one participant commenting:

I guess what’s going to be really interesting is in the coming months, as I start to go out more in public after COVID-19, and I cough, and what people’s reaction is going to be … It’s going to be real tricky in public … You can’t stop coughing, and the reaction of the people around you.

### Sub-theme—Respected

The majority of participants had experiences where they felt people understood and respected what the pandemic meant for them, specifically that they understood and complied with in-person contact restrictions participants deemed necessary. One participant demonstrated this by saying:

For the most part, my friends definitely understood what I was doing had to be done. No one would voluntarily go into isolation if they didn’t have to.

In addition, some participants felt that the pandemic may even increase society’s relatability to them through an increased awareness of health and infection control, issues people with CF face on an ongoing basis. One participant commented:

Now you guys know what it’s like to have CF. Now guys know what it’s like to have to worry about people who’ve got the flu, because now it’s, welcome to my world … Now maybe you’ll understand us a bit better now … Welcome to the uncertainty of the world. Welcome to the uncertainty of health.

### COVID-19 specific: Theme 3—Social support

This theme refers to social support within a COVID-19 specific context. The theme of social support is derived from the majority of participants identifying social support as an outcome and an enhancer of social connectedness, during the pandemic. Participants described social support in the form of instrumental (tangible) social support and emotional social support. Two sub-themes are derived.

### Sub-theme—Proximal social support

The majority of participants reported availing of instrumental or emotional social support from family or friends during the pandemic. Instrumental support included provision of essential supplies, such as groceries, to enable reduced in-person contact. This is demonstrated by one participant, who stated:

A lot of family and friends have been supportive of isolation measures … Like in family, checking in that I’ve got everything I need, so that they can help me keep as isolated as possible.

Emotional support comprised of expressions of care and concern from family and friends. In particular, participants described how receiving emotional social support strengthened friendship bonds. One participant stated:

Especially being in isolation, I’ve realized who my real friends are, and they were the ones that check in on me. It’s also strengthened my friendship with one of my friends.

### Sub-theme—Distal social support

Many participants reported accessing instrumental social support from the community, specifically neighbours, medical practitioners, schools, colleges, and employers during the pandemic. Community social support enabled them to work and attend to medical and childcare needs, all while reducing in-person contact. The provision of instrumental social support from the community promoted a sense of togetherness, gratefulness and community spirit. One participant articulated this:

Like at the hospital, the pharmacy have been mailing out drugs. Tomorrow, I’ve got a medical appointment, and that’s over the phone, so it’s been really well thought out. Even the local pharmacy … They’re actually bringing medications out to my car … I had my flu shot while I was sitting in my car at the doctor’s … It’s really built or reinstated some of the community that’s been lost I think.

## Discussion

Findings contribute to a limited pool of qualitative research exploring social connectedness within both a general CF and COVID-19 specific context. Aligning with belongingness theory (Kohut, 1984) and previous research (e.g., Begun et al., 2018; Hatchcock, 2012; Toth, 2016), participants articulated an innate need for social connectedness, social connectedness challenges posed by CF, and the value of social connectedness in optimizing the mental and physical health of adults living with CF.

Our findings support existing research outside CF which suggests several mechanisms linking social connectedness with health, for example, the influence of social network health norms on health behaviour, and social connectedness enabling access to practical and emotional support to help buffer stress (Larrabee Sonderlund et al., 2019; Uchino, 2006; Uchino et al., 1996, 2013). This is particularly significant in a cohort of people with a chronic and complex disease, who face elevated rates of anxiety and depression symptoms and a demanding daily treatment burden (Quittner et al., 2014a; Eakin et al., 2011). It follows that social connectedness-based conversations, assessments and interventions as part of routine CF healthcare are recommended, with the goal of enhancing social connectedness where required.

There are a range of evidence-based techniques to improve social connectedness, most of which have been researched within an older adult setting (e.g., increased personal contact, group participation, support to develop personal skills, various forms of therapy; O’Rourke et al., 2018). Our findings highlight the importance of fostering relationships with a virtuous cycle of receiving social support and experiencing a sense of social connectedness. Future research to design and evaluate specific social connectedness interventions for adults living with CF is recommended. Interventions that aim to identify levels of social connectedness and enhance it where necessary could prove useful in optimizing CF health outcomes.

Consideration should be given to online communication platforms when designing social connectedness interventions. The ability to enhance social connectedness via online communication has unsurprisingly been a talking point for researchers since the growth of social media during the past decade, coupled with the in-person contact restrictions of COVID-19 (Hwang et al., 2021). For some vulnerable groups in this pandemic, such as the elderly (Armitage & Nellums, 2020), harnessing online technologies to promote social connectedness has proved a challenge. The ability to enhance social connectedness through online communication hinges upon a range of factors, for example, socioeconomic status, online literacy, level of disability and method of online communication (Masoud et al., 2021). Research does however demonstrate that if people have access and ability, attaining a sense of belonging and social connectedness online is indeed possible and associated with improved mental health (Armitage & Nellums, 2020; Clark et al., 2018). Given the CF community’s pre-pandemic familiarity with online platforms, and the range of infection control and time advantages it offers inside and outside of COVID-19, it seems logical that we must utilize this skill.

Adults with CF demonstrated resilience during COVID-19, adapting well under adverse circumstances or significant stress (American Psychology Association, 2021). Adaptation and positivity are considered important dimensions of psychological resilience (Fletcher & Sarkar, 2013), with social connectedness a suggested mechanism for resilience (Dang, 2014; Denz-Penhey & Campbell Murdoch, 2008). Resilience is a trait previously identified within the CF community (Mitmansgruber et al., 2016), and future research exploring social connectedness within the broader psychological concept of resilience could offer useful insights. It is conceivable that the CF community entered this pandemic armed with applicable experience of living with CF infection control measures, in-person contact restrictions and online communication which has contributed to their resilience during COVID-19.

Aligning with previous research, our study highlights the power of empathy and positive human relationships in promoting psychological health for adults with CF during this pandemic (Nobili et al., 2021). When empathy and respect were received from proximal relationships and society in general, a sense of togetherness and community was experienced. CF healthcare teams play an important role in nurturing their human relationships and connection with those living with CF, as well as raising community awareness of the importance of empathy and social connectedness during an era of uncertainty and contact restrictions.

## Strengths & limitations

Lincoln and Guba (1985) substitute the notion of validity for trustworthiness in qualitative research. In this study, the researcher conducting participant interviews and data analysis has extensive experience as a CF social worker, providing contextual knowledge and expertise to promote rigour (Fetterman, 2010). Additionally, the entire study, including data analysis, was overseen by a team of senior researchers with extensive experience in both CF research and clinical care. A summary of study findings was provided to participants prior to publication, with no subsequent changes made.

There are several study limitations which must be acknowledged due to potential impact upon findings and generalizability. First, this was a small-scale study with theoretical saturation unmet due to time constraints. Second, the study took place within Western Australia, a state with a specific COVID-19 context which may differ to other geographical areas. Third, there is no way to be sure that the participants were not experiencing depression or mental illness, related or unrelated to the COVID-19 situation. Last, the people most likely to be medically stable and volunteer for study participation have the potential to be those with a high level of social connectedness. Results therefore may not reflect those struggling with low levels of social connectedness in the cohort. Moreover, a large majority of participants are employed and live with someone. These are potential social connectedness protective factors, and again may not be representation of the most vulnerable people.
